# Technology-assisted cognitive-behavior therapy delivered by peers versus standard cognitive behavior therapy delivered by community health workers for perinatal depression: study protocol of a cluster randomized controlled non-inferiority trial

**DOI:** 10.1186/s13063-023-07581-w

**Published:** 2023-08-25

**Authors:** Atif Rahman, Abid Malik, Najia Atif, Huma Nazir, Ahmed Zaidi, Anum Nisar, Ahmed Waqas, Maria Sharif, Tao Chen, Duolao Wang, Siham Sikander

**Affiliations:** 1 Department of Primary Care and Mental Health, Institute of Population Health, University of Liverpool, Waterhouse Building, Block B First Floor, 1-5 Dover Street, Liverpool, L69 3GL UK; 2 Health Services Academy, Islamabad, Pakistan; 3 Human Development Research Foundation, Islamabad, Pakistan; 4 University of York, York, UK; 5 Liverpool School of Tropical Medicine, Liverpool, UK

**Keywords:** Perinatal depression, Psychosocial intervention, Cognitive-behavioral therapy, Thinking Healthy Programme, Digital intervention, Technology, Mental health

## Abstract

**Background:**

The lack of trained mental health professionals is a key barrier to scale-up of evidence-based psychological interventions in low and middle-income countries. We have developed an app that allows a peer with no prior experience of health-care delivery to deliver the cognitive therapy-based intervention for perinatal depression, the Thinking Healthy Programme (THP). This trial aims to assess the effectiveness and cost-effectiveness of this Technology-assisted peer-delivered THP versus standard face-to-face Thinking Healthy Programme delivered by trained health workers.

**Methods:**

We will employ a non-inferiority stratified cluster randomized controlled trial design comparing the two formats of intervention delivery. A total of 980 women in the second or third trimester of pregnancy with a diagnosis of Major Depressive Episode, evaluated with the Structured Clinical Interview for DSM-V Disorders (SCID), will be recruited into the trial. The unit of randomization will be 70 village clusters randomly allocated in a 1:1 ratio to the intervention and control arms.

The primary outcome is defined as remission from major depressive episode at 3 months postnatal measured with the SCID. Data will also be collected on symptoms of anxiety, disability, quality of life, service use and costs, and infant-related outcomes such as exclusive breastfeeding and immunization rates. Data will be collected on the primary outcome and selected secondary outcomes (depression and anxiety scores, exclusive breastfeeding) at 6 months postnatal to evaluate if the improvements are sustained in the longer-term. We are especially interested in sustained improvement (recovery) from major depressive episode.

**Discussion:**

This trial will evaluate the effectiveness and cost-effectiveness of a technology-assisted peer-delivered cognitive behavioral therapy-based intervention in rural Pakistan. If shown to be effective, the novel delivery format could play a role in reducing the treatment gap for perinatal depression and other common mental disorders in LMIC.

**Trial registration:**

The trial was registered at Clinicaltrials.gov (NCT05353491) on 29 April 2022.

**Supplementary Information:**

The online version contains supplementary material available at 10.1186/s13063-023-07581-w.

## Background

The prevalence of perinatal depression in low- and middle-income countries (LMICs) is estimated to range from 23.8 to 27.8% [1, 2]. South Asia stands out as the region where the rates of perinatal depression are among the highest in the world, and in Pakistan, the prevalence of perinatal depression has been reported to be 30 to 37% [3]. Untreated perinatal depression is of concern not only because of its effect on maternal health but also from the effect that impaired maternal role fulfillment has on the mother-infant bonding and child care and the long-term impact on the infant’s physical and cognitive development [4]. In Pakistan, children of mothers with depression are 1.5 times more likely to be underweight and 1.4 times more likely to be stunted [5]. In UK, the value of total lifetime costs of perinatal depression has been estimated to be over USD $100,000 per woman with the condition [6], but in Pakistan, due to the association with infant malnutrition, the relative impact is likely to be greater.

A number of studies have demonstrated that evidence-based psychosocial interventions for perinatal depression can be effectively delivered by non-specialist health professionals [7]. In Pakistan, we developed and evaluated the Thinking Healthy Programme (THP) designed to be delivered by community health workers. The program incorporated cognitive behavior therapy (CBT)-based techniques focusing on the mother’s mental health and its effect on her relationship with the infant and significant others and inter-session practice activities to help the mother and family to problem-solve [8]. A large randomized controlled trial showed that THP more than halved the rate of depression compared with usual care and led to significant improvements in women’s functioning and disability, and the intervention was effective in the poorest populations [9, 10]. In 2015, the THP was incorporated into the World Health Organization’s flagship Mental Health Gap Action Programme (mhGAP) for global dissemination [11].

A key barrier to scale-up of the THP in LMICs is the lack of trained health professionals to deliver the intervention. Even where health workers are available, they are over-burdened, which makes it difficult to sustain the program beyond pilot sites. In recent studies, peers (women from the same localities with no prior experience of health-care delivery) have been found to be feasible delivery agents of the Programme [12, 13]. However, longer-term effectiveness of the peer-delivered intervention tends to decrease [12], possibly as a result of what Chambers and colleagues [14] refer to as a voltage drop, i.e., the intervention loses some degree of its potency or fidelity when moving from efficacy to effectiveness in the real world, and program drift, i.e., the intervention deviates from its manualized or implementation protocols [14]. Applying a technological solution to this challenge, we have developed a technology-assisted version of THP which allows the peers to deliver the Thinking Healthy Programme sustainably without the need for extensive training and supervision. Our approach does not rely solely on technology but builds counseling skills of peers in order to maintain human-contact and empathy, while ensuring the active therapeutic ingredients are delivered to fidelity and at the right dose with the help of a peer-operated app. We envisage this innovation will assist in scale-up of the THP through all types of lay-workers, making the intervention accessible to the most under-served populations.

## Aim and hypotheses

The aim of this trial is to evaluate the effectiveness and cost-effectiveness of the technology-assisted Thinking Healthy Programme (THP-TA). Our primary hypothesis is THP-TA delivered by peers will be non-inferior to standard THP delivered by community health workers in improving remission rates in women with perinatal depression at 3 months postnatal.

We will test several secondary hypotheses both at 3 and 6 months postnatal. We hypothesize that compared to the standard THP, THP-TA will be effective in (a) reducing symptoms severity of perinatal depression at 3-months postnatal, (b) reducing the symptoms severity and improving remission rates of perinatal anxiety at 3 and 6 months postnatal, (c) reducing the maternal disability at 3 and 6 months postnatal, (d) improving maternal quality of life at 3 and 6-months postnatal, (e) improving recovery rates of perinatal depression at 6-months postnatal, and (f) will be cost-effective compared to THP in treating perinatal depression across the pregnancy to 6 months’ postnatal period. Other maternal and infant outcomes include rates of exclusive breast-feeding, rates of immunization, parental time spent playing with the infant and rates of contraception use among women at 3 and 6 months postnatal. As part of the secondary outcomes we will evaluate the process of implementation and fidelity of THP-TA delivered by peers for treating perinatal depression. Lastly, we will also explore mediational pathways of THP-TA in improving perinatal mental health outcomes. Acceptability of the THP-TA intervention will be explored in an embedded qualitative study from the perspective of the end-users and their significant family members and delivery agents [15–17].

## Methods

### Trial settings

We used the SPIRIT checklist when writing our report (Supplementary file 1).

The trial will be conducted in rural areas of Kallar Syeddan, Gujar Khan, and Rawalpindi, within Rawalpindi District of Punjab, Pakistan. The rural economy is based largely on subsistence farming, semi-skilled or unskilled laboring, or low-paid government service in nearby cities. The female literacy rate is about 46.88% and the infant mortality rate about 53 per 1000 live births [18, 19]. The study area consists of three subdistricts with 19 Union Councils (UCs) or rural administrative units, comprising several villages. These villages are served by community health workers called lady health workers (LHWs). The primary role of the LHWs is to provide health education and basic maternal and child health care through monthly home visits. Each LHW is responsible for about 250 households and keeps a register of every new pregnancy in her catchment area.

### Design

We will employ a non-inferiority stratified cluster randomized controlled trial design comparing technology-assisted THP delivered by peers with standard THP delivered by LHWs. We use a non-inferiority design because standard THP delivered by LHWs has demonstrated strongly positive effects on perinatal depression in a previous trial [9].

### Randomization

The study area comprises 70 village clusters situated in 19 rural administrative units (Union Councils) where program implementation is approved by the District Health Office. Each village cluster has a population ranging from 2400 to 3600 and is served by two or three LHWs. All 70 village clusters will be randomly allocated in a 1:1 ratio to the intervention and control arms. The stratification will be at the level of the Union Council (UC), while a village cluster (VC) will form the unit of randomization. The reason for choosing village cluster as the unit of randomization is to minimize contamination/spillage between trial participants as the perinatal depression intervention will be delivered within community-based households. The intervention and control village clusters are geographically distinct and the chance of intervention cluster participants communicating on a regular basis with control cluster participants is very small.

Randomization will be done before the participants are recruited. Research teams responsible for identifying, obtaining consent, and recruiting trial participants will be blind to the allocation status. This will help to minimize post-randomization recruitment bias. Randomization codes will be generated via a permuted-block randomization method and stratified by Union Council. Block sizes will vary at two, four, and six. Allocation of clusters will be carried out by an independent statistician based at Liverpool using SAS PROC Plan.

### Participants

**Fig. 1:**
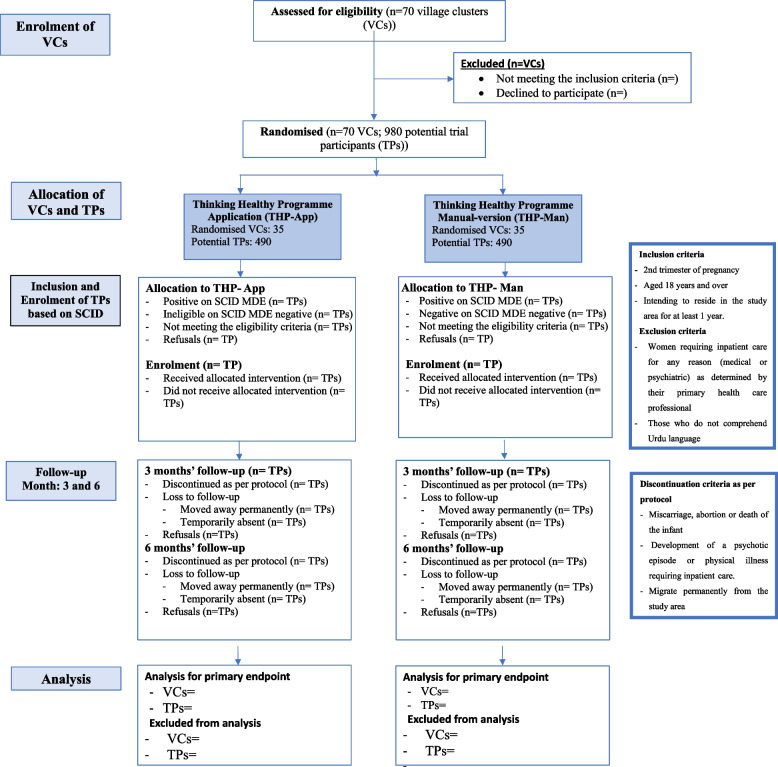
Trial flow diagram

**Fig. 2:**
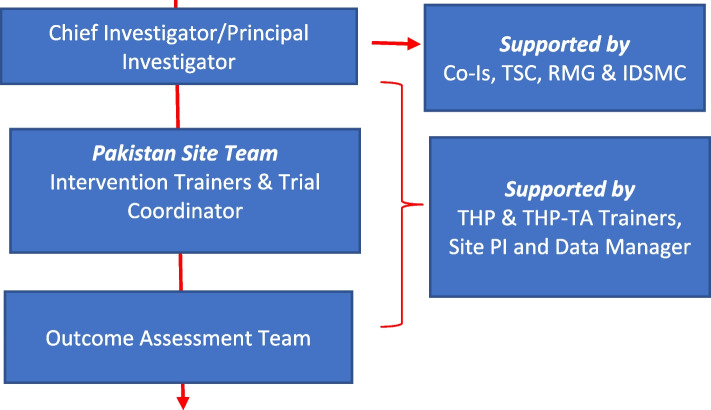
Trial governance structure

All pregnant women living in the participating village clusters who are on the registers of the LHWs, will be assessed for eligibility. All potentially eligible women will be evaluated for current major depressive episode by researchers trained in using the structured clinical interview for Diagnostic and Statistical Manual of Mental Disorders (DSM)-IV diagnosis, which is a semi-structured diagnostic interview [20] that has been widely used in cross-cultural epidemiological and treatment studies of prenatal and postnatal depression [21]. We have translated and culturally adapted the section for major depressive episode into Urdu using a rigorous procedure [22] and have used it extensively in our previous studies [9]. All pregnant women with current major depressive episode who give informed consent to participate will be recruited into the trial. As this is a pragmatic trial, we do not have restrictive inclusion/exclusion criteria. Figs. 1 and 2 presents the CONSORT flow diagram delineating recruitment procedures.

### Inclusion criteria

Eligible participants will be pregnant women with major depressive episode in their second or third trimester, aged 18 years and above, who intend to stay in the study area for at least 1 year.

### Exclusion criteria

Women requiring inpatient care for any reason (medical or psychiatric) as determined by their primary health care professional or those who do not comprehend Urdu language will be excluded.

### Informed consent and confidentiality issues

Community meetings with village elders, local counselors, and primary health care doctors will be conducted in each Union Council, during which information about trial activities will be shared. Village cluster-level consent will be obtained from the District Health Office responsible for providing primary health care to Rawalpindi District. At the individual participant level, informed written consent will be obtained before screening and baseline assessment. The procedure will be undertaken by trained researchers.

Information about the rights of trial participants, including confidentiality and voluntariness, will be provided. No personal information will be associated with the data of the respective participants; all data will be assigned a study identification number, and only this number will be used for the study assessments. Identification data will be kept in a password protected computer, which will only be accessible to the data manager. No identifying personal characteristics will be used in any publication or presentation. Participation in the study will be completely voluntary and participants will retain the right to refuse answers to any questions that they do not feel comfortable with or to withdraw from the study at any point in time. Such withdrawal will have no negative effect on the health care the participant receives through the LHWs or at any other health care institution, and it will not limit any other rights for the participant or her baby. The consent will be sought in Urdu after at least 24 h of receiving the information about the trial. Prior to signing the informed consent form, the trial participants will have the opportunity to carefully review the written consent form and ask questions regarding the study.

Wherever possible, the participant’s family will be advised of the need for confidentiality so that the family understands the conditions under which information will be shared. In addition to this, the research team will reiterate confidentiality to family members if any family member would be present at screening or at any other outcome assessment. The research team will be instructed not to divulge information to other family members and will only do so if the trial participant would be at risk to herself or others. While obtaining the consent, the participants will be explicitly informed that any incidence of domestic violence required by law to be reported to the primary care physician may be reported to the authorities without the permission of participants. Furthermore, any clinical worsening (e.g., lowering of mood or suicidal ideation) may result in referral for treatment and the research team could potentially disclose this information to individuals outside of the study or to family members. If the research team believes that the trial participant is at risk of harm to herself or to someone else, the research team will report this to the area’s primary care physician without the consent of the participant. The consent forms will need to be signed by the trial participants and the researcher who will take consent. A copy of the information sheets and consent forms will be left with the participants. A record of age, depression score, and reasons for refusal will be maintained for those who do not consent.

Prior to recruitment, ethical approval will be sought from Research Ethics Committees of Human Development Research Foundation, Pakistan, University of Liverpool, UK, and National Bioethics Committee, Pakistan. Ethical approval will also be sought before the commencement of the trial.

### Monitoring for adverse events and discontinuation criteria

Previous trials of the THP indicate the intervention is associated with negligible adverse events. However, a register will be maintained of potential adverse events, defined as any unfavorable signs, symptoms, or disease associated with the use of a treatment. For this study, specific adverse events include (a) death of the mother due to any cause; (b) suicide attempt; (c) domestic violence against the participant; (d) infant abuse or neglect; (e) stillbirth, miscarriage, or death of infant; (f) hospital admission of participant due to any physical or psychiatric problem; and (g) clinical deterioration.

The detection of potential adverse events will be done by the outcome assessment team (at 3 months and 6 months postnatal) and by the intervention delivery agents (LHWs or peers) throughout the period of intervention delivery in both arms. All these personnel will be trained in detecting the adverse events using questionnaires and mood ratings in each session. Talking about feelings or sensitive topics may be difficult and cause emotional upset in some participants. The intervention delivery agents and assessors in both arms will be trained in counseling skills, which will help them to console the participants if such a situation arises. Equally, delivery agents may also be emotionally affected by listening to emotionally disturbed participants. This will be addressed in supervision.

Trial participants will be discontinued from the study at any time-point in case of miscarriage, abortion, or death of the infant, as determined by the health personnel. They will be offered support from a trained professional. Participants who develop a psychotic episode or physical illness or serious medical condition requiring inpatient care (determined by primary care professional) will also be discontinued and referred to a specialist facility for management. Participants who withdraw their consent or migrate permanently from the study area will also discontinue the study. Discontinuation from the intervention (i.e., THP-TA and THP) will mean that all trial activities will stop for these participants (including follow-up assessments and data collection) leading to discontinuation from the study altogether (and these will not be included in the analyses).

Participants experiencing domestic violence will be offered specialist social services at a primary or tertiary facility for support and management but not discontinued from the study. Similarly, in case of infant abuse or neglect, the participants will be offered specialist social services at a tertiary facility but continue to participate in the trial. The primary care physician will be informed of all such cases.

The current trial is sponsored by the University of Liverpool. The University of Liverpool holds insurance against claims for compensation for injury caused by participation in a clinical trial. The conduct of the trial as described in this protocol is covered under the University of Liverpool’s Clinical Trials insurance policy.

### Masking

Due to the nature of the intervention, it is not possible to mask participants and delivery agents to treatment allocation. However, outcome evaluation will be masked, as all outcome measures will be administered by trained outcome assessors, who are non-residents of the study area, independent of the intervention and masked to treatment allocation of the participants. Furthermore, participants will be instructed not to disclose to the outcome assessors how they receive the intervention. During all assessments, the primary outcome measure (SCID) will always be completed first to minimize the risk of bias in the event of unmasking and, if it occurs, the point of unmasking will be recorded. Sensitivity analyses will be carried out to assess the effect of unmasking on the measurement of primary outcome. The intervention and outcome assessment teams will not have any interactions during the trial since there will be separate locations and administrative management. The assessment teams will be told that they are evaluating two interventions and that there is genuine equipoise about which one is better.

### Baseline assessments

Baseline assessment will include the following information: (i) age in years, (ii) marital status, (iii) obstetric history, (iv) educational attainment, (v) employment status, (vi) perceived social support, and (vii) incidence of domestic violence in the last 3 months. The baseline assessments will take place at a venue of the participants’ choice—either at her own home or at the LHWs’ facility called the Health House or at any other household in the community.

## Interventions

### Standard Thinking Healthy Programme

The standard Thinking Healthy Programme (THP) is an evidence-based manualized intervention targeting women with perinatal depression in low socioeconomic settings [8]. It aims to improve health outcomes in the women and their children through the adaptation and integration of CBT techniques into the routine work of community health workers [8]. The CBT techniques include guided discovery using illustrated brief vignettes, behavioral activation, and problem solving [8]. Non-specific techniques include empathic listening, psychoeducation, and promoting social support from key family members for the mother in negotiating challenges during the perinatal period. The intervention employs these techniques to improve outcomes in three areas: maternal well-being, mother-infant interaction, and relationship with significant others [9]. The intervention identifies resources within the family and community to address modifiable risks to mental health such as interpersonal conflict. The intervention consists of 8 core sessions starting in the second or third trimester of pregnancy and continuing to 3 months postnatal. The THP manual with a step-by-step guidance on delivery is downloadable from the WHO website in a number of languages [23].

Mothers in the standard treatment group will receive the THP through 40 specially trained LHWs (i.e., one for each village cluster). The LHWs are trained in a 5-day classroom-based workshop (approximately 4–5 h daily), conducted by specialist trainers using a previously trialed technology assisted cascaded training model [24]. The training format is based on interactive discussions and role-plays, including those dealing with difficult situations. The training topics cover communication skills, underlying theoretical concepts, techniques employed in the intervention, and their step-by-step implementation. Discussions also covers the use of job-aids related to the intervention (e.g., health calendars) and elaborate on the structure and methodology of supervision.

### Technology-assisted Thinking Healthy Programme

We employed the Human Centred Design (HCD) [21, 22] to develop the technology-assisted version of the Thinking Healthy Programme (THP-TA). Details of the development process are reported elsewhere [25]. In summary, our HCD approach involved (a) involving users throughout design and development, (b) conducting an iterative process with frequent feedback loops, (c) addressing the whole user-experience, and (d) incorporating multidisciplinary skills and perspectives in the design team.

The standard THP manual’s guidance and step-by-step instructions for delivering each session [23] were converted into narrative scripts by a panel of experts. An artist converted the characters into “avatars” (graphic image representing each character) which were used to voice the narrative scripts. These culturally appropriate animated avatars representing the therapist, peer, depressed mothers, and other key family members enacted scenarios based on actual issues derived from our qualitative research and experiences of delivering the intervention in the community. Each session consists of several interactive scenarios, with pause buttons and instructions, allowing the health worker, women, and their family-members to discuss each scenario in the context of their own lives and share potential helpful solutions to problems identified. The active ingredients and the core content in the technology-assisted version remain identical to the standard THP. The final technology-assisted version consists of a multimedia android-based app that can be used by a variety of health workers to facilitate program delivery. The prototypes were subjected to extensive user-testing before finalization.

In this arm, the delivery agents are peers. Peers are lay women from the same locality, without any health training background who have shown an interest or desire to help and support other women within their community [26]. All peers are identified and recruited from the local community through community-based key informants. As much of the therapy content is automated and has in-built mechanisms to ensure fidelity, the supervision and training does not require a specialist. Training will be provided by a trained non-specialist who has practiced intervention-delivery for 3 months under supervision. The training is focused on improving communication skills of the peers, giving them a basic understanding of the key ingredients of the intervention, and practicing the operational elements of the app. As less practice is required in delivering the key elements of the intervention, the training is briefer (3 days). Monthly peer-supervision will be conducted.

### Evaluating competency of delivery agents

In order to ensure the fidelity of the intervention, assessments will be conducted using the World Health Organization’s resource for trainers and supervisors called EQUIP (Ensuring Quality in Psychological Support) [27]. The EQUIP assessment tools namely the THP competencies and ENACT FHS-A (ENhancing Assessment of Common Therapeutic factors, Foundational Helping Skills for Adults) will be used following the THP training and over the trial period [28]. The 11-item THP competencies form and the 15-item ENACT FHS-A, rate intervention-specific skills, and the foundational helping skills respectively on a four-point Likert scale ranging from level 1 (any unhelpful behavior) to level 4 (all basic skills plus advanced skills). Assessments will be conducted using the EQUIP standardized role plays that will be linguistically and culturally adapted for use in the Pakistani context. Role plays will be assessed by an independent assessors trained in using the tools and on the THP. These assessments will ensure that the intervention is delivered with a high level of quality in order to achieve the intended effects.

## Outcomes

**Fig. 3:**
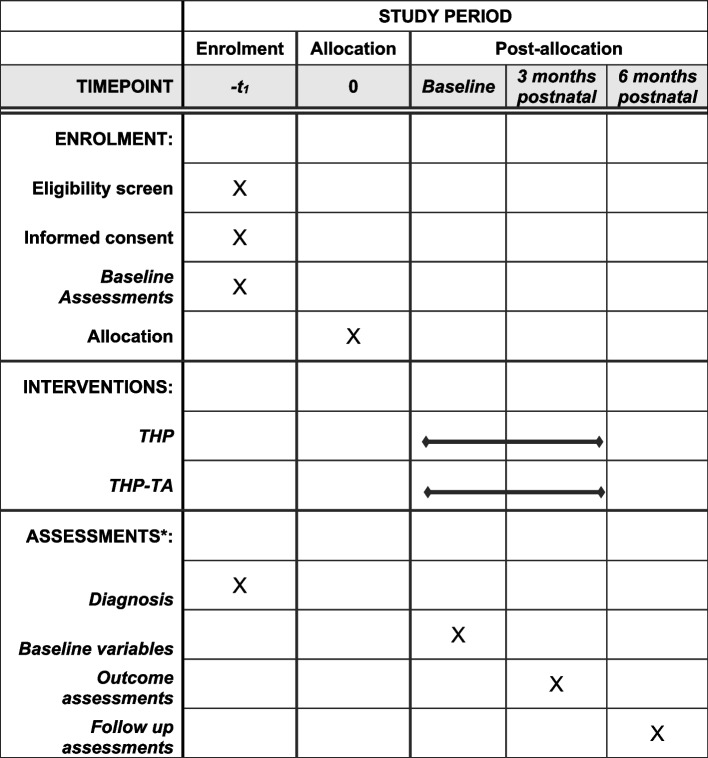
Schedule of enrolment, interventions, and assessments. Asterisk (*) symbol indicates the following: please see Table 1 for details on assessments

The study outcomes have been detailed in Table 1 and Fig. 3.

**Table 1:** Schedule of study assessment activities for main study

Outcomes	Measures/assessed by	3rd month postnatal		6th month postnatal	Translations and validity carried out	Description of the outcome measure
		**Primary outcomes**	**Secondary Outcomes**	**Secondary outcomes**		
**Perinatal depression**	SCID current MDE module	% of non-MDE cases	**x**	% of non-MDE cases	Cross-culturally validated and used in study settings	Diagnostic interview for case non-case
**Perinatal depression severity**	PHQ-9	**x**	total mean scores	total mean scores	Validated and used in study settings	DSM-IV criteria-based depression screening tool; 9 items; 0–27 score; categories of severity
**Perinatal anxiety**	GAD-7		Total mean scores	Total mean scores	Translated and used in study setting	DSM-IV criteria based generalized anxiety disorder screening tool; 7 items; 0–21 score; categories of severity
			% of mild, moderate, severe cases	% of mild, moderate, severe cases		
**Maternal quality of life**	EQ-5D-3L		Severity levels	Severity levels	Translated and used in study setting	Presents results on a descriptive system describing different aspects of health: mobility, self-care, usual activities, pain/discomfort and anxiety/depression. Each dimension describes problems in different aspects of health across three categories: no problems, some problems, and extreme problems
**Infant nutritional status** **Breastfeeding practices**	24-h recall WHO Breastfeeding Practices tool		% of exclusive breast feeding	% of exclusive breast feeding	Translated and used in study setting	
**Cost-effectiveness**	Client service receipt inventory		**x**		Translated and used in study setting	CSRI is used to collect data pertaining to healthcare services used, details and costs of medications, and wider health service utilization and economic costs. It comprises of five sections including client information, accommodation and living circumstances and costs, employment, household income, and details on paid and unpaid healthcare services

### Primary outcome

Our primary outcome is defined as remission from major depressive episode at 3-months postnatal measured with the Structured Clinical Interview for DSM-IV Disorders (SCID) Major Depressive Episode (MDE) Module. SCID is a semi-structured interview for the diagnoses of psychiatric disorders [20]. It has been cross-culturally adapted for assessing depression among women during pregnancy and in the postpartum period [21] and has been extensively used in the study settings [9].

### Secondary outcomes

We collect data on several secondary outcomes at 3 and 6 months postnatal.

#### Symptoms of depression

We will use the Patient health questionnaire (PHQ-9) [29] that scores the nine DSM-IV symptom-based criteria for depression on a four-point Likert scale from not having the symptom at all to having it nearly every day, over the last 2 weeks. The score for each item is summed to arrive at a total score. The cut-off point of 10 is selected as the most accurate value for the detection of depression and has a high positive predictive value for the diagnosis of depressive disorder [29] and has been used extensively in Pakistan [30].

#### Symptoms of anxiety

We will use the Generalized Anxiety Disorder 7-Items (GAD-7) that scores the seven, DSM-IV symptom-based criteria for generalized anxiety disorder on a four-point Likert scale from not having the symptom at all to having it nearly every day, over the last 2 weeks [31]. The score for each item is summed to arrive at a total score. This tool also has been extensively used in the study settings [32].

#### Disability

The WHO disability assessment schedule 2.0 (WHO-DAS) is a 12-item questionnaire for measuring functional impairment over the last 30 days [33]. In addition, two items assess the number of days the person was unable to work in the past 30 days. It gives a total disability score, quality adjusted life years, and number of days out of work. WHO-DAS has been validated for international use [33] and used in our previous studies [9, 12, 13].

#### Quality of life

We will use the EuroQol measure (EQ-5D-3L) to assess health-related quality of life among the trial participants. It is a valid and reliable scale which has been extensively used to quantity health status across 5 dimensions: mobility, self-care, usual activities, pain/discomfort, and anxiety/depression. Responses on the EQ-5D can also be used to generate quality-adjusted life years (QALYs) for cost-effectiveness and cost-utility analyses.

#### Infant-related secondary outcomes

Exclusive breastfeeding will be based on 24-h maternal recall using the WHO definition of exclusive breastfeeding. We will also collect data on immunization, family planning/use of contraception, time spent in play, and episodes of diarrheal and acute respiratory tract infections among infants. We have used these measures in our previous studies [9].

#### Service use and costs

Service use will be measured using Client Service Receipt Inventory (CSRI) [34]. It is used to collect data pertaining to healthcare services used, details and costs of medications, and wider health service utilization and economic costs. It comprises of five sections including client information, accommodation and living circumstances and costs, employment, household income, and details on paid and unpaid healthcare services.

### Sustained improvement

Data will be collected on the primary outcome and selected secondary outcomes (depression and anxiety scores, exclusive breastfeeding) at 6 months postnatal to evaluate if the improvements are sustained in the longer-term. We are especially interested in sustained improvement (recovery) from major depressive episode.

We will explore possible mechanisms of intervention-effects by employing mediational analysis with a number of a priori constructs hypothesized to mediate THP-TA effects. We hypothesize that the intervention effects of the THP-TA are driven by two non-specific ‘active’ ingredients (a) social support and (b) empathic relationship with the therapist and three specific “active” ingredients: (a) patient activation, (b) problem solving, and (c) cognitive restructuring (Table 2). Measures for each of these constructs is given as follows.

**Table 2:** Mediators exploring the mechanism of change due to THP-TA

Construct	Measure	Description
**Perceived social support**	Multidimensional Scale of Perceived Social Support	It comprises of 12 items and three subscales related to social support from family, friends, and significant other
**Empathy and compassion**	Empathy scale for lay therapists	The level of compassionate care delivered by peers to the intervention recipients will be assessed using a 12-item The Empathy Scale for Lay Therapists. This scale has been developed by a panel of psychiatrists, psychologists, and experts by experience
**Patient activation**	The PREMIUM Abbreviated Activation Scale	It comprises of 5 items and is based on the longer Behavioral Activation for Depression Scale and has been found to be reliable and valid for use in a similar population
**Problem solving**	Problem-solving confidence subscale of the Problem-solving Inventory	It comprises of 11 items and assesses perceived confidence for use of problem-solving skills gained from a psychotherapeutic intervention
**Cognitive restructuring**	Cognitive restructuring subscale of the frequency of actions and thoughts scale	The use cognitive restructuring skills among the study participants will be assessed using the cognitive restructuring subscale of the frequency of actions and thoughts scale. This subscale comprises of three items

### Social support

We will use the Multidimensional Scale of Perceived Social Support (MSPSS) to assess the perceived levels of social support among the intervention recipients [35]. It comprises of 12 items and three subscales related to social support from family, friends, and significant other. This scale has been translated and validated for use in this study setting [35].

### Empathic relationship

The level of compassionate care delivered by peers to the intervention recipients will be assessed using a 12-item The Empathy Scale for Lay Therapists. This scale has been developed by a panel of psychiatrists, psychologists, and experts by experience.

### Behavioral activation

We will use The PREMIUM Abbreviated Activation Scale (PAAS), to assess levels of behavioral activation in the sample. It comprises of 5 items and is based on the longer Behavioral Activation for Depression Scale and has been found to be reliable and valid for use in a similar population [36].

### Problem solving

Problem solving skills among the study participants will be assessed using the Problem-solving confidence subscale of the Problem-solving Inventory. It comprises of 11 items and assesses perceived confidence for use of problem-solving skills gained from a psychotherapeutic intervention [37].

### Cognitive restructuring

The use cognitive restructuring skills among the study participants will be assessed using the cognitive restructuring subscale of the frequency of actions and thoughts scale. This subscale comprises of three items and has yielded good cross-cultural validity [38].

## Fidelity of the intervention

Data will be collected in both arms on both quantitative and qualitative indicators of fidelity of the interventions using previously validated methods [39]. Quantity indicators will include (a) number and duration of sessions delivered—information will be collated from peer logbooks, which will record basic information about the session, such as date, time, and duration of the session; (b) number of participants who complete the treatment and have a planned discharge; and (c) number of participants who are treatment failures or need referral to mental health specialist services for any other reason. Quantitative indicators will include competency of delivery agents and satisfaction with treatment. Competency of the delivery agents will be assessed using the THP competencies form and the ENACT scale [28, 40] and satisfaction using indigenously developed, close-ended Likert scale type questions.

## Data collection and management

The data will be collected electronically on tablets and uploaded to the cloud server on daily basis from the field. The electronic form will be designed with all possible skips and logics as to minimize the efforts of the data collection team and to increase their efficiency. Quality checks for consistency, accuracy, missing data, and other irregularities will be conducted weekly. The issues will be addressed timely and feedback will be given to the assessors during weekly research staff meetings. All the data will be backed up daily on cloud as well as on different local storages. Data will be anonymized before being shared with the co-investigators outside of the institution. Data stored will be password protected and will not be accessible to anyone but authorized personnel.

Data stored at the server will be checked for missing or out of range values (via range checks) and checked for consistency within participants over time. Any suspect data will be returned to the site team in the form of data queries. Data query forms will be produced at the server from the trial database and sent electronically. The site will respond to the queries providing an explanation/resolution to the discrepancies and return the data query forms to the server. The forms will then be filed along with the appropriate audit logs and the appropriate corrections will be made on the database.

## Economic evaluation

Both cost-utility and cost-effectiveness analyses will be performed in this trial. Economic costs will be collected from a societal perspective, with a time horizon of 6 months. The primary outcome of the study is remission from major depressive episode at 3 months. However, quality-adjusted life years (QALYs) will also be used as an outcome measure in the analysis because they contain both longevity and quality of life, allowing comparisons across different diseases and treatment modalities. Quality of life will be estimated using EQ-5D-3L at baseline and end of scheduled follow-up, and it will be converted to utility value using tariffs from the Pakistan EQ-5D-3L value set [41].

CSRI will be used to record services used across the entire duration of the study [34]; covering the period of pregnancy until 6 months postnatal including healthcare services used, details and costs of medications, and wider health service utilization and economic costs. Specifically, data will be collected on two resource uses: (i) intervention costs (include all the resources required to organize and implement the intervention through peers and (ii) other healthcare service costs (traditional, religious, hospitals, medicines, care by family members) incurred during the trial period (may also include drugs and medical supplies, patient out-of-pocket expenses incurred from the individual’s time spent seeking for services for mental health care, treatment, and travel expenses).

The economic analysis will be conducted and reported according to the CHEERS Statement [42]. Analysis of the economic data will be based upon intent-to-treat principles. We will present the cost and health outcome data descriptively by summarizing the total cost and its component among each group and compare the different between groups. Afterwards, bootstrap techniques will be used to estimate a cost-effectiveness acceptability curve, displaying the likelihood that the intervention could be cost-effective. Cost-effectiveness planes and acceptability curves were used to illustrate uncertainty around incremental cost-effectiveness ratios (ICERs). Further sensitivity analyses will be conducted to assess the robustness of results and the cost-effectiveness after the trial in the future real world.

## Sample size and statistical analysis

Sample size estimation is based on the primary outcome measure, remission from a major depressive episode elicited by the Structured Clinical Interview for DSM-IV. In our initial randomized trial of THP [9], there was a 75% remission rate in the intervention arm. For non-inferiority trials, it is vital to select a relevant limit for the possible difference between arms that will lead to rejection of the hypothesis of non-inferiority [43]. In this trial, as both arms involve active treatment based on THP, we assume 75% remission rates in both arms at 3 months postnatal and set the limit to − 10%, which would still be considered to be of public health significance [44]. As this is a cluster randomized trial, we set an intracluster correlation (ICC) of 0.005 to allow for between-village cluster correlation. Alpha is set to 0.025, resulting in a one-sided 97.5% confidence interval or two-sided 95% confidence interval for of the assessment of non-inferiority. Allowing for 70 village clusters randomized with a 1:1 allocation ratio and 14 depressed participants per village cluster, and 20% loss to follow-up, a total of 980 participants will be required to detect non-inferiority for the primary outcome at 3 months postnatal with a power of 87.2%.

Primary outcome will be analyzed using a generalized linear mixed model (GLIMM). It will be based on the intention-to-treat population. Non-inferiority will be declared if the lower limit of one-sided 97.5%CI is larger than the non-inferiority margin of − 10%. The model will have a binomial distribution and identity link function with the treatment (intervention vs control) as a fixed effect, baseline measurement of SCID score as covariate, and cluster as random effect. In addition, adjusted GLIMM model analysis will be performed with the pre-specified covariates measured at baseline being added into the above GLIMM model, which will be identified and listed in the statistical analysis plan. The unadjusted and adjusted risk differences between intervention and control group in the primary outcome together with its 95% confidence intervals (CIs) will be derived from the GLIMM models. The main conclusion will be drawn from the unadjusted analysis. In addition, subgroup analysis of primary outcome will be performed on the above pre-specified covariates.

Analysis of secondary binary outcomes with repeated follow-up measurements will be analyzed using GLMMIX models. The model will have a binomial distribution and logit link function with the treatment (intervention vs control), visit (3 and 6 months), interaction between treatment and visit as fixed effects, baseline measurement of outcome as covariate, and cluster and subject as random effects. The odds ratios between intervention and control group together with its 95% CIs at each visit will be derived from the GLIMM models. Analysis of secondary continuous outcomes with repeated follow-up measurements will be performed using GLMMIX models with normal distribution and identity link function. The models will have the treatment (intervention vs control), visit (3 and 6 months), interaction between treatment and visit as fixed effects, baseline measurement of the outcome variable as covariate, and cluster and subject as random effects. The mean differences with 95%CIs between intervention and control at each visit will be derived from the GLMMIX model. Other statistical analyses may be performed if deemed necessary.

Primary data analyses will be based on the intention-to-treat principle. The per-protocol analyses will also be performed as supplemental analysis. All analyses will be detailed in the statistical analysis plan which will be finalized before the un-blinding of the study. All statistical analyses will be performed using SAS 9.4. The trial results will be reported following the CONSORT guidelines for cluster-randomized trials.

Exploratory mediation analyses will be conducted in a multilevel structural equation modeling framework using the MPlus software. For this, we will test a 2–1-1 mediation model, where mediation is hypothesized at the level of clusters. This model is found to be appropriate because the intervention condition is randomized at the level of clusters rather than individuals. The mediator variables and PHQ-9 scores available at the level of individuals will be transformed into cluster level mean scores to model higher level variance and then group mean centered scores for lower-level variance.

## Oversight and monitoring

### Governance structure of the trial

Figure 2 shows the governance structure of the trial. The CI/PI is Atif Rahman (AR) who has overall responsibility of the trial. The PI has extensive experience in conducting epidemiological studies and randomized controlled trials evaluating mental health interventions delivered by non-specialist/lay health workers in Pakistan. He will be supported by co-investigators, trial steering committee, research management group, and an independent data safety and monitoring committee (IDSMC).

### Trial steering committee (TSC)

The trial steering committee will consist of an independent chairperson plus the existing research management group members who are experts in the field of developing and testing perinatal mental health interventions, health economics, and biostatistics. The role of the TSC is to provide overall supervision for THP-TA trial and provide advice through its independent chairperson. The ultimate decision for the continuation of the trial lies with the TSC. The TSC will first convene in February 2022 and will meet regularly during the trial period of 30 months (at least annually).

### Independent data and safety monitoring committee (IDSMC)

The independent data and safety monitoring committee consists of an independent chairperson and independent members who are experts in the fields of public health, reproductive health and maternal wellbeing and medical statistics.

The IDSMC will be responsible for reviewing and assessing recruitment, interim monitoring of safety and effectiveness, trial conduct, and data management. The IDSMC will first convene in February 2022 and will meet regularly during the trial (at least annually). No formal interim analysis is planned.

Further information regarding conduct and membership of oversight committees will be maintained in the folder with the following documents:

- Research management group terms of reference.
- Trial steering committee terms of reference.
- Independent data and safety monitoring committee terms of reference.

### Protocol amendments

In the event of any amendments to the protocol, the following process will be adhered to the following.

#### Notifying sponsor and funder

Any proposed changes will first be communicated to the sponsor and funder for their review and approval.

#### Communication with principal investigator (PI) and centers

Upon receiving the necessary approvals, the PI will be notified, and a copy of the revised protocol will be sent to the PI and all involved centers. The PI will be responsible for adding the updated document to the Investigator Site File.

#### Documentation of deviations

Should there be any deviations from the original protocol, they will be fully documented using a breach report form, outlining the nature of the deviation, the reasons behind it, and any corrective actions taken.

#### Update in the clinical trial registry

Any changes to the protocol will be reflected in the clinical trial registry, ensuring full transparency and alignment with regulatory requirements.

## Discussion

This study evaluates the effectiveness and cost-effectiveness of a technology-assisted intervention delivered by peers. The Lancet Commission on Global Mental Health reviewed the use of technology to facilitate training as well as delivery of psychological interventions and highlighted this as a major area for future research to assist scale-up of programs such as THP and help reduce the treatment gap for mental disorder [45]. This is supported by systematic reviews on technology-assisted mental health interventions in LMIC which underlines the potential role of technology in addressing the treatment gap for mental disorder in LMIC [46, 47]. Naslund and colleagues reviewed evidence on the use of mobile, online, and other remote technologies for treatment and prevention of mental disorders in LMICs [46]. Of the 49 studies identified, most were preliminary evaluations of feasibility and acceptability of technology for supporting clinical care and educating health workers, mobile tools for facilitating diagnosis and detection of mental disorders, technologies for promoting treatment adherence and supporting recovery, online self-help programs for individuals with mental disorders, and programs for substance misuse prevention and treatment.

In Pakistan, we have previously developed technology-assisted tools to (a) provide training of community health workers in the THP [24] and (b) provide caregiver skills training to parents of children with developmental disorders [48]. This preliminary work has demonstrated the feasibility of technology to assist in the training and delivery of psychosocial interventions in the rural Pakistani community. Building on this work and the global evidence for technology-assisted approaches, we have developed a technologically-enhanced version of THP which will assist the peers in the delivery of the THP. To our knowledge, this is the first innovation of its type. We envisage the automated features built into the innovation will allow the intervention to be delivered to fidelity and prevent voltage drop and program drift which are major barriers to scale-up of task-shifted psychosocial interventions.

A key gap identified in the use of technologies for mental health is the lack of rigorous studies to evaluate effectiveness, assess costs, and carefully consider potential risks of digital technology interventions for mental health. This problem is not confined to LMIC—a plethora of apps and other technologies have been marketed in developed countries that claim to provide treatments for anxiety, depression, schizophrenia, and addictions. However, very few have been subjected to randomized trials that are mandatory for any new treatment before it is released for public consumption [49]. We hope the results of this study will stimulate further innovation and research in this important area that can be a game-changer in addressing the treatment gap for common mental disorder globally.

## Trial status

The recruitment for the trial began in June 2022. The protocol version number is 3, dated 14 September 2022. The recruitment for the trial is ongoing and ended on May 30, 2023. The interventions are currently being offered to the trial participants, and two more waves of assessments will be conducted. Due to delay in revision of the final draft of the manuscript, we were not able to submit the manuscript well in advance of recruitment procedures.

### Supplementary Information

**Additional file 1.** Reporting checklist for protocol of a clinical trial.

## Data Availability

Data associated with this manuscript is available upon request to the corresponding authors.
